# Synthesis of Porous Boron-Doped Carbon Nitride: Adsorption Capacity and Photo-Regeneration Properties

**DOI:** 10.3390/ijerph16040581

**Published:** 2019-02-17

**Authors:** Xiang Guo, Lei Rao, Peifang Wang, Lixin Zhang, Yuxiong Wang

**Affiliations:** 1 Key Laboratory of Integrated Regulation and Resource Development on Shallow Lakes, Ministry of Education, College of Environment, Hohai University, Nanjing 210098, China; gx2015@hhu.edu.cn (X.G.); zhangjia00047@163.com (L.Z.); 171305020063@hhu.edu.cn (Y.W.); 2 College of Mechanics and Materials, Hohai University, Nanjing 21100, China

**Keywords:** adsorption, photocatalytic, regenerate, carbon nitride, B_2_O_3_

## Abstract

Carbon nitride (CN) with improved adsorption–degradation capacity was synthesized using B_2_O_3_ and CN via calcination. The pollutant removal capacity of this B_2_O_3_/CN (B-CN) was studied by a powder suspension experiment and added into concrete to evaluate the adsorption and degradation of methylene blue (MB). The characterizations of all samples indicate that B_2_O_3_ significantly affects CN, e.g., by increasing the CN specific surface area to 3.6 times the original value, extending visible light adsorption, and narrowing the band gap from 2.56 eV to 2.42 eV. Furthermore, the results show that B-CN composite materials have a higher MB-removal efficiency, with the adsorption capacity reaching 43.11 mg/g, which is about 3.3 times that of pristine CN. The MB adsorption process on B2-CN is mainly via electrostatic attraction and π–π interactions. In addition, B-CN added into concrete also has good performance. After five adsorption–degradation cycles, B-CN and photocatalytic concrete still exhibit a good regenerate ability and excellent stability, which are very important for practical applications.

## 1. Introduction

In the past decades, photocatalysis by semiconductor materials has been extensively used for the removal of toxic organic and inorganic water and air pollutants [1,2,3,4]. These materials are required to have properties such as strong light absorption ability, suitable band gap, and redox potential, and they should also be stable, non-toxic, inexpensive, and easily machinable into desired shapes. Furthermore, the application of photocatalytic materials is limited by issues such as a short reaction time, low light intensity, inefficient pollutant interception and second pollution, which are the disadvantages of photocatalytic material in the improvement of the removal efficiency of water pollutants. Therefore, an adsorption–degradation system and adsorbent photocatalyst materials may enhance the removal efficiency to treat polluted water.

Among the potential catalysts, carbon nitride (CN) has been considered one of the most promising metal-free semiconductor materials [5]. The CN preparation process is simple: usually, CN is prepared by the thermal polymerization of carbon- and nitrogen-containing organic molecules such as urea, melamine, thiourea, dicyandiamide, and cyanamide. Otherwise, it has a good photocatalytic effect, forbidden bandwidth of about 2.7, and high efficiency in removing organic pollutants, heavy metals, and viruses [6,7,8]. Thus, the CN-based photocatalysts and their applications have potential in solving energy and environmental issues in the future. On the other hand, the photocatalytic efficiency of CN under visible light has been found to be low, and it absorbs only 0.1% of irradiated visible light [9]. Furthermore, the high recombination rate of photogenerated electron–hole pairs limits the applicability of CN [10]. Therefore, to improve the properties of CN, including photocatalytic efficiency, adsorptivity, and solar light utilization, many methods have been developed, such as doping with metallic [11] or nonmetallic elements [12], coupling with other materials [13], and changing the morphology [14]. The adsorptivity of CN is also an important factor that influences the removal efficiency, and it has thus attracted considerable attention [15,16]. Generally, the adsorption capacity of a given adsorbent is strongly dependent on both its porous microtexture [14,17] and surface chemical property [18]. Therefore, some studies have increased the specific surface area using the acid treatment of CN or its precursor [19]. However, the acid treatment requires acid form, washing, drying, and calcining, making the process more complicated. Most importantly, this method did not significantly increase the adsorption capacity of CN. Therefore, a novel method with simpler steps to enhance the adsorption–degradation ability of CN is necessary.

Boron-doped polymeric CN as metal-free catalysts have been studied to improve the photocatalytic property, but their adsorption property has not been investigated. Yan et al. [20] reported the utilization of B_2_O_3_ in the preparation of B-doped CN photocatalytic material. They infer that the element B could replace the H on the amino group of carbon nitride’s structure. The BCN has good photocatalytic efficiency for MO (Methyl Orange) and RhB (Rhodamine B). Sagara et al. [21] demonstrated that B-doped carbon nitride was prepared from BH_3_NH_3_ and melamine. Then, Au, Ag or Rh as a co-catalyst was coated on the surface of g-C_3_N_4_ and B-doped g-C_3_N_4_ by magnetron sputtering. They found that B-doped g-C_3_N_4_ coated with Rh has the highest photocurrent response under solar light irradiation, its photocurrent being about 10 times larger than that of original g-C_3_N_4_. Lu et al. [22] prepared B-doped carbon nitride from thiourea and boric acid by calcination at high temperature. The optimized B-g-C_3_N_4_ photocatalyst exhibited an excellent removal efficiency of UO_2_^2+^ and good photocatalytic stability.

There are many common B sources, such as BH_3_NH_3_ [23,24], Boron pieces [25], and B2O3 [26]. It has been considered that the structure of B2O3 is almost perfectly suited for a glassy network model, in which the boron atoms are surrounded by three oxygen atoms, whereas oxygen atoms are surrounded by two boron atoms [27]. Therefore, B_2_O_3_ is often used as a dopant to modified different materials [20,28]. Therefore, we too were inspired to use B_2_O_3_ to prepare a novel CN material with enhanced adsorption and photocatalytic capacities. In this study, B_2_O_3_ was utilized not only as a dopant but also as a structure modifier. This suggests that introducing boron into the CN structural framework will enhance the specific surface area and narrow its band gap to absorb more visible light.

Here, we report a series of boron-doped CN for improving the adsorption capacity and photocatalytic activity. To our knowledge, this is the first time that solid melting-state is used to modify CN and create pores, thus increasing the specific surface area. Methylene blue (MB) dye was selected as the model contaminant for the adsorption-photocatalytic experiments due to its presence in wastewaters and industries effluents. The results of experiments showed that the B_2_O_3_/CN (B-CN) series of materials exhibit superior performance than CN. Furthermore, according to the process of adding B-CN materials into concrete, we found that the compound substrate yielded good adsorption-fixation efficiency. Therefore, this work opened a low-cost way for material modification and photocatalytic concrete, which is very important for practical applications.

## 2. Experimental

### 2.1. Sample Preparation

CN: Melamine as a precursor was placed into a ceramic crucible and covered with a ceramic lid. Then, it was heated in a program-controlled-atmosphere furnace from room temperature to 550 °C at a heating rate of 5 °C/min and maintained at 550 °C for 2 h. When the furnace cooled to room temperature naturally, the material was ground and CN was collected.

Repeatedly calcined CN: the CN powder was calcined once more using the above-mentioned procedures. The resultant CN was denoted as Re-CN.

**Scheme 1:**
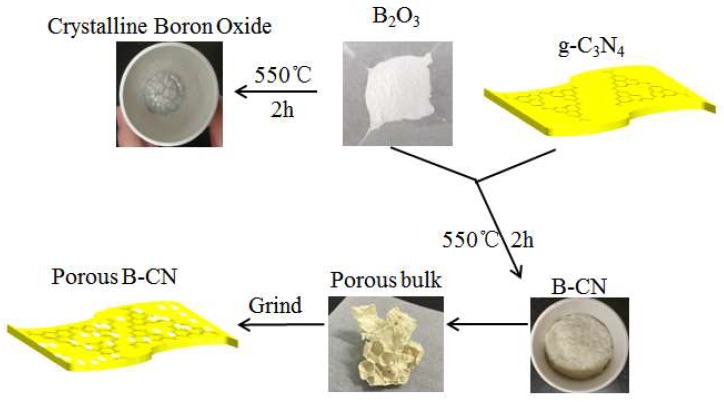
Preparation of B-CN powders.

Composite of CN and boron oxide (B_2_O_3_): The CN and boron oxide were mixed into different weight proportions (3:1, 1:1, and 1:3) and then ground evenly. Then, the three different composite powders were placed one at a time into a ceramic crucible. Then, the samples were heated to 550 °C at a heating rate of 5 °C/min and maintained at 550 °C for 2 h. When the furnace was cooled to the room temperature, the powders were washed with water and ethanol several times. For convenience of description, the composite material samples are hereinafter abbreviated as B1-CN (CN/B_2_O_3_ = 3:1), B2-CN (CN/B_2_O_3_ = 1:1), and B3-CN (CN/B_2_O_3_ = 1:3). The preparation of B-CN is presented in Scheme 1.

**Figure 1:**
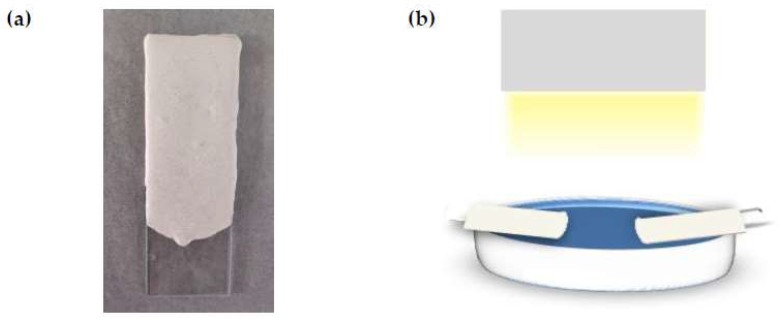
The concrete specimens samples after cured 28 days (**a**) and photocatalytic experiment setup (**b**).

Photocatalytic concrete: The concrete specimens were prepared from white cement, metakaolin, fine sand, CN (or B-CN) and water, which used a 1:0.5:2.6:0.5:1 ratio of the above components (Blank concrete specimens (BCS) with no photocatalytic materials). The photocatalytic concrete specimens (PCS) samples employed in the photocatalytic experiments were prepared on glass surfaces and measured 75 mm × 25 mm × 1.5 mm. The base samples (as Figure 1a shows) were cured for 28 days at ambient conditions (25 °C and 50% relative humidity).

### 2.2. Characterization

The structural analysis of the samples was carried out by X-Ray diffraction (XRD, Rigaku Ultima III, Tokyo, Japan) and recorded in the 2θ range of 5°–80° with a scan rate of 0.02°/0.4 s using a Bruker AXS D8 system (Bruker, Billerica, MA, USA) equipped with a Cu Kα radiation source (λ = 0.15406 Å), in which the X-ray tube was operated at 40 kV and 40 mA. UV-Vis diffuse reflectance spectra (DRS) were obtained on a UV-visible (UV-Vis) spectrophotometer (PerkinElmer, Waltham, MA, USA) with BaSO_4_ as the reference. Low-temperature N_2_ adsorption/desorption measurements (Brunauer–Emmett–Teller (BET) method) were carried out using a Micromeritics ASAP 2020 system (Micromeritics, Norcross, GA, USA) at −196 °C, following degassing of all samples at 120 °C for 2 h. X-ray photoelectron spectroscopy (XPS) measurements were performed on a Thermo Scientific ESCALAB 250 instrument (Thermo Fisher Scientific, Waltham, MA, USA) with Al Kα source. The sample morphology of the different photocatalyst materials was examined using a scanning electron microscope (SEM, Hitachi, Tokyo, Japan) and a transmission electron microscope (TEM, JEM-2100, JEOL, Tokyo, Japan).

### 2.3. Adsorption and Photocatalytic Degradation of MB

In the adsorption experiment, 30 mg of different samples were dispersed in 100 mL of different concentrations of MB solution (5 mg/L, 10 mg/L, 15 mg/L, 20 mg/L, 25 mg/L, 30 mg/L) in 180 min. Otherwise, the photocatalytic experiment was carried out in the similar way. In dark reaction, 30 mg of different samples was added in 100 mL of 20 mg/L MB solution to reach adsorption saturation. Then, photocatalytic activities of all samples were assessed under irradiation with a 300 W xenon lamp visible light (λ > 400 nm) to trigger the photocatalytic reaction. During different adsorption and irradiation durations, 1.5 mL of solution were collected from the reaction vessel and centrifuged before analysis.

However, the photocatalytic concrete experiment was carried out as shown in Figure 1b. Place the PCS inclined into the 60 mL MB solution (10 mg/L) and keep the semi-exposure state. In addition, other experimental procedures are consistent with the powder experiment, except for no stirring. The MB adsorption ability of PCS photocatalytic activity was studied in dark for 1 h to reach adsorption saturation. Then, put them under irradiation with a 300 W of xenon lamp with visible light (λ > 400 nm) to assess the photocatalytic activity.

The removal of the MB was monitored using a UV-vis spectrophotometer (Shimadzu, Kyoto, Japan) at 664 nm. The percentage of removal of MB is calculated by using the following equation:

(1) $$R_{t}=\frac{\left(C_{0}-C_{t}\right)}{C_{0}}*100\%,$$

where *R_t_* is the removal rate at time *t* after commencing the adsorption and photocatalytic degradation process, and *C*_0_ and *C_t_* are respectively the initial concentration and the concentration at time *t*.

The adsorbed quantity of different samples is calculated as follows:

(2) $$Q_{e}=\frac{\left(C_{0}-C_{e}\right)V}{m},$$

where *Q_e_* is the adsorbed quantity of samples at the equilibrium moment of adsorption and desorption. *C*_0_, *C_e_*, *V* and *m* are the initial concentration, concentration at time *t*, initial volume of MB, and quantity of adsorbent, respectively.

### 2.4. Kinetic and Adsorption Isotherm Model

The adsorption kinetics is an important characteristic, which defines the adsorption efficiency. Therefore, the kinetic models were used to fit the experimental data and determine the kinetics of the adsorption process. The linear expressions of pseudo first-order kinetic model and pseudo second-order kinetic model were shown as follows:

(3) $$Pseudo\;first\;order\;kinetic\;model:ln\left(Q_{e}-Q_{t}\right)=lnQ_{e}-k_{1}t,$$

(4) $$Pseudo\;second\;order\;kinetic\;model:\;\frac{t}{Q_{t}}=\frac{1}{k_{2}Q_{e}^{2}}+\frac{t}{Q_{e}},$$

where *Q_t_* and *Q_e_* are the adsorbed quantity of samples at time *t* and at equilibrium moment, respectively. *k*_1_ and *k*_2_ are the rate constants of pseudo first order kinetic model and pseudo second order kinetic model.

The Langmuir and Freundlich isotherm model are two mathematical models for estimating the adsorbate affinity, surface property, and adsorption capacity of adsorbent. The Langmuir model assumes monolayer coverage and that all adsorbent sorption sites are the same. While the Freundlich isotherm model assumes that the coverage is multilayer and all the adsorption sites are heterogeneous. The two equations of isotherm model were shown as follows:

(5) $$Langumuir:\;\frac{C_{e}}{Q_{e}}=\frac{1}{Q_{m}K_{L}}+\frac{C_{e}}{Q_{m}},$$

(6) $$Freundlic:\;lnQ_{e}=\frac{1}{n}lnC_{e}+lnK_{F}.$$

In the two equations, *Q_e_* and *Q_m_* represent the equilibrium adsorption capacity and the maximum adsorption capacity, respectively. Otherwise, *C_e_* stands for the pollution concentration at adsorption equilibrium. *K_L_*, n and *K_F_* are all contents.

## 3. Results and Discussion

### 3.1. Characterizations of CN and Boron-Doped Carbon Nitride B-CN

**Figure 2:**
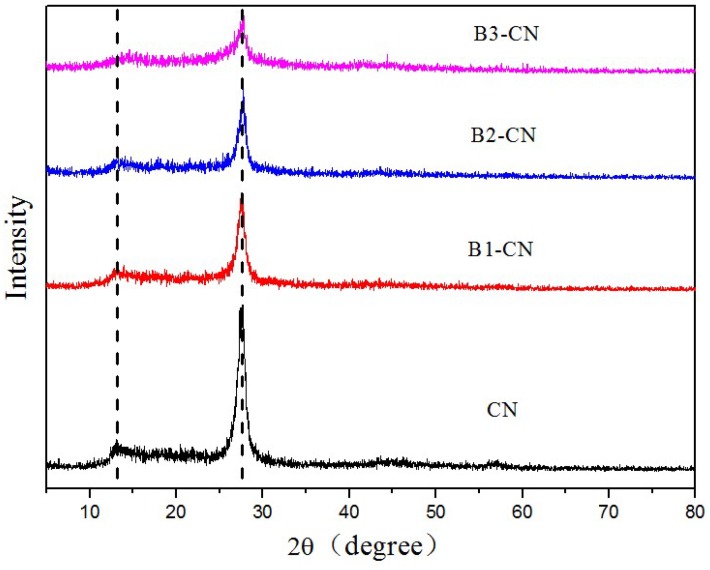
XRD patterns of pure CN and B_2_O_3_/CN composite materials with different CN/B_2_O_3_ proportions (CN/B_2_O_3_ = 3:1 (B1-CN), 1:1 (B2-CN), 1:3 (B3-CN)).

To compare the CN, Re-CN and B-CN series materials, the XRD patterns were recorded. In Figure 2, the XRD patterns of different samples show two characteristics peak at 27.4° and 13.1°. The two diffraction peaks are consistent with (100) and (002) planes of CN (JCPDS 87-1526), which relate to the in-plane structural packing motif and the interlayer stacking of aromatic systems [29], respectively. To compare the influence of B_2_O_3_ on CN, the XRD patterns of the CN composite with different B_2_O_3_ were recorded and shown in Figure 2. When B_2_O_3_ is added, the diffraction peaks of B1-CN correspond to lower intensities than those of pure CN. With an increasing amount of B_2_O_3_, the intensities corresponding to the diffraction peaks at 27.4 °C and 13.1 °C decreased evidently. Furthermore, no obvious new peaks appeared in the B-CN XRD patterns.

**Figure 3:**
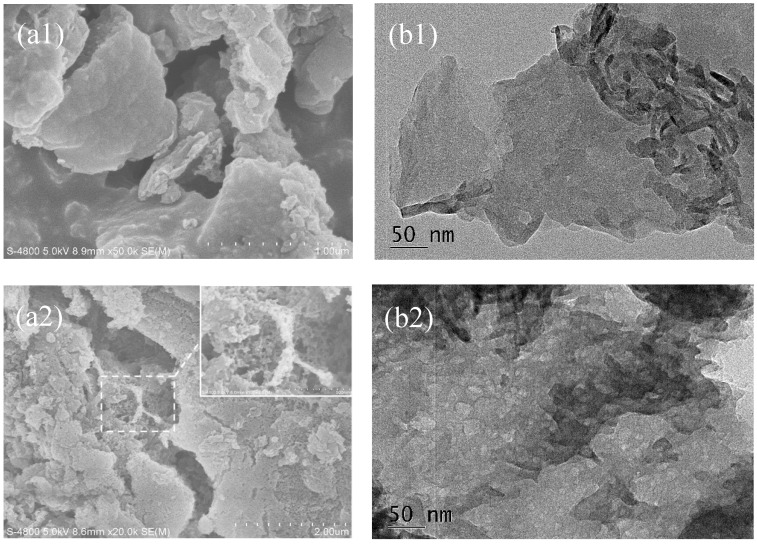
SEM (**a**); micrographs of pure CN (**a1**); and B2-CN (**a2**); TEM (**b**); micrographs of CN (**b1**); and B2-CN (**b2**).

The surface morphologies and microstructure of the CN and B_2_O_3_/CN composite material were examined by SEM and TEM. Figure 3a1,b1 show the structure of pure CN that has a 2D-lamellar stacked structure. As the representative of the B_2_O_3_/CN composite materials, B2-CN has different microstructures from that of pure CN. Many highly unconsolidated structures with pores were found on irregularly shaped lamellar structures, as shown in Figure 3a2. The TEM microstructure of Figure 3b2 showed that these pores are irregularly distributed in the layered structure of the CN. These pores may be formed owing to the melting state of the B_2_O_3_ during the heating process wrapped CN; then, gases such as air or ammonia gas were produced and caused the pore formation on the surface of CN.

**Figure 4:**
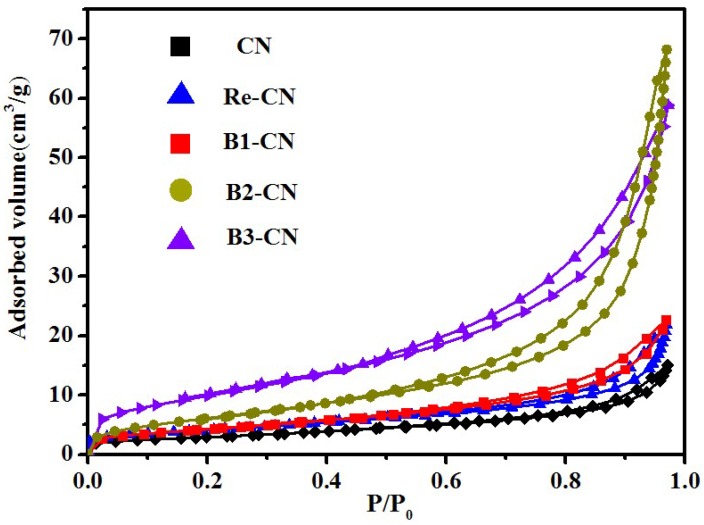
Nitrogen adsorption/desorption isotherm profiles for CN (black); Re-CN (blue); B1-CN (red); B2-CN (yellow); and B3-CN (purple).

The specific surface area and porosity of CN are two crucial factors affecting the adsorption and photocatalytic reactions. As shown in Figure 4, the representative N2 adsorption-desorption isotherms of bulk CN and CN/B_2_O_3_ series are of type IV. According to the IUPAC type, the isothermal adsorption desorption curves exhibited the type “H3” hysteresis loop (0.7 < P/P_0_ < 0.97: CN, Re-CN, B1-CN; 0.5 < P/P_0_ < 0.97: B2-CN, B3-CN). The small hysteresis loops of CN and Re-CN located at high P/P_0_ are caused by the aggregation of 2D-lamellar structure. By contrast, the B-CN series materials exhibited an obvious hysteresis loop without limiting the adsorption at high P/P_0_, indicating the presence of more pores formed by B_2_O_3_ during calcination. Furthermore, details regarding the specific surface areas, average pore diameters, and pore volumes of different samples are summarized in Table 1. Comparisons of CN and Re-CN show that the repeated calcination increased the specific surface area, pore size, and pore volume values. Furthermore, all values increased obviously when B_2_O_3_ was mixed with CN and calcined. Among the B-CN series, B2-CN showed the highest specific surface area, average pore size, and pore volume, which is in good agreement with the characteristics of surface pores in SEM and TEM photographs.

**Figure 5:**
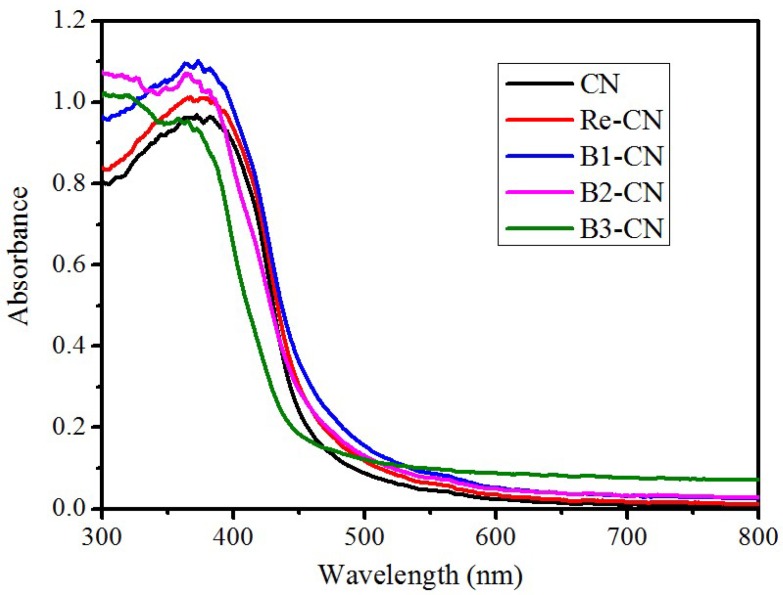
Diffuse reflectance spectra of CN, Re-CN, and CN/B_2_O_3_ series composite materials.

The DRS of the pure CN, Re-CN and B_2_O_3_/CN composite materials are shown in Figure 5. All samples exhibited good absorption properties at 300–450 nm. Re-CN, B1-CN, and B2-CN have better light absorption ability than CN and the maxima absorption wavelength were all observed at 365 nm. After B_2_O_3_ addition and calcination, an increase in the UV−vis light absorption over the entire wavelength range is observed. However, when the B_2_O_3_ content increased, the optical absorption feature of B_2_O_3_/CN decreased before 500 nm, and then increased. Furthermore, compared to pure CN, the B_2_O_3_/CN composite materials have a broader absorption range and better light absorption properties, which may be beneficial to the improvement of photocatalytic activities [30].

**Figure 6:**
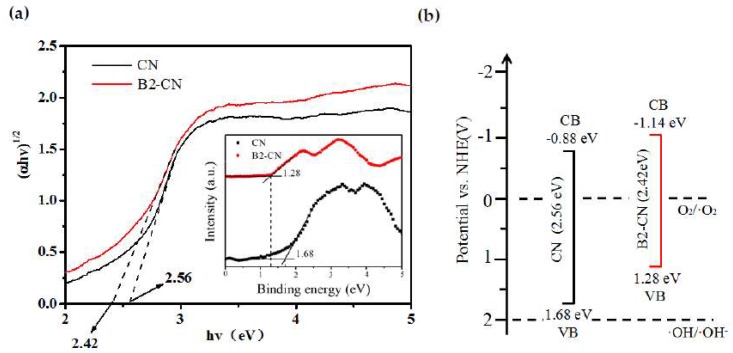
(**a**) indirect electron transitions and VBXPS (inset) of CN and B2-CN; (**b**) the determined CB and VB edges of CN and B2-CN.

The optical absorption properties of the materials were determined through estimation of the optical band-gap as outlined in Equation (7):

(7) $$Ahv=C{\left(hv-Eg\right)}^{n},$$

where *C* is a constant and *A*, *Eg*, *h*, and *v* represent the absorption coefficient, band-gap energy, Planck constant, and incident light frequency, respectively. Values of 1/2 and 2 for the constant n correspond to direct and indirect transitions, respectively. Based on the calculations and plots of (Ahv)^1/2^ versus hν presented in Figure 5a, the band gaps of CN and B2-CN are determined as 2.56 and 2.42 eV, respectively. This probably is attributed to the structure defects formed in the samples treated at melting state of the B_2_O_3_ during the heating process wrapped CN, which improve the optical absorption of materials. Furthermore, in order to analyze the band structure clearly, XPS measurements were carried out (Figure 6a insert graph). The VB potentials were 1.68 eV (CN) and 1.28eV (B2-CN). The conduction bands (CB) are calculated to be −0.88 (CN) and −1.14 (B2-CN). As is shown in Figure 6b, the VB potentials of B2-CN shifts positively leading to more oxidation of the holes and increasing photo-oxidation capacity, and the VB potentials of CN and B2-CN are all not more positive than E^0^ (OH/OH^−^ = +1.99 eV vs. NHE) [31]. This demonstrates that ·OH could not be directly generated from the oxidation of OH^−^ or H_2_O. Furthermore, the CB potential of B2-CN is more negative than E^0^(O_2_/O_2_·^−^ = −0.046), indicating that O_2_ can trap electrons to form O_2_^−^ and H_2_O_2_. These species are vital for photocatalytic degradation of MB [32].

**Figure 7:**
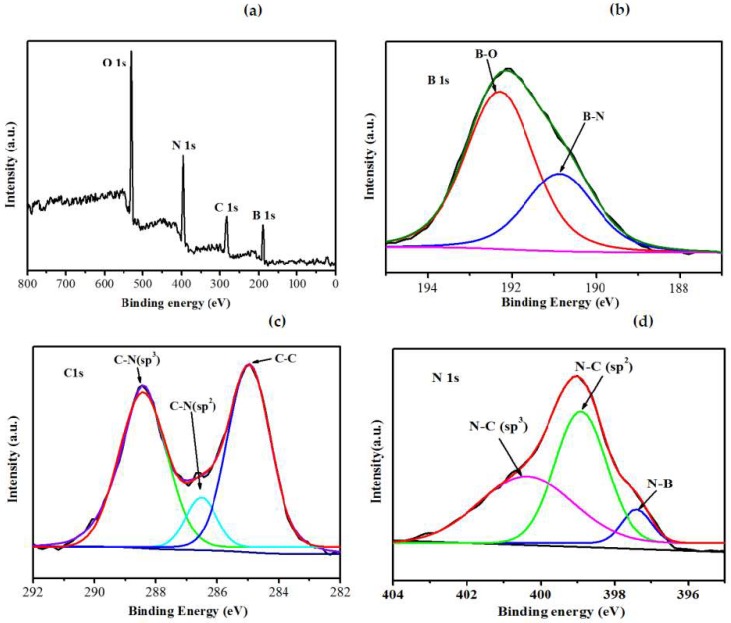
XPS survey spectra of (**a**) product and the deconvoluted spectra of (**b**) B1s, (**c**) C1s, (**d**) N1s.

X-ray photoelectron spectroscopy (XPS) was performed to investigate the surface elemental composition and chemical states of B2-CN. Figure 7a shows the XPS spectra of B 1s, N 1s, C1s, and O 1s. The B1s (b) spectrum of B-CN shows two components at 192.3 eV and 190.8 eV, corresponding to B-O and B-N, respectively [25]. This indicates that some boron atoms are introduced into the CN frame. The C 1s spectrum of CN shows three species with binding energies of 288.4 eV, 286.6 eV, and 284.9 eV. The peak at 284.9 eV is typically assigned to graphitic sp^2^ C = C bonds, while the peak at 288.4 eV is attributed to the C-N-C bond [20]. In addition, an accompanying weak peak at 286.6 eV is ascribed to C-NH_2_ [14]. As shown in Figure 7d, the N 1s spectrum of CN can be deconvoluted into three different peaks with binding energies at 397.5 eV, 398.7 eV, and 400.5 eV. The peak at 398.7 eV is typically attributed to the sp^2^ nitrogen of triazine of the triazine rings in C—N = C, whereas the peak located at 400.5 eV corresponds to the bridging N atoms (N-(C)_3_), and that at 397.5 eV is ascribed to N-B [25].

**Figure 8:**
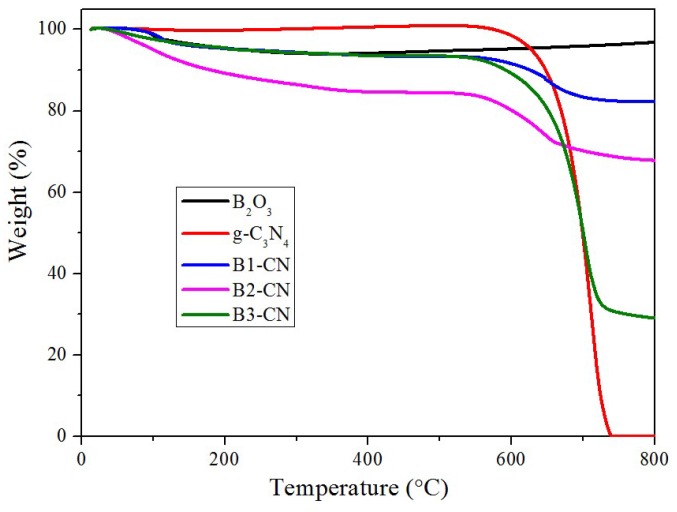
The thermo gravimetric curves of B_2_O_3_, CN, and CN/B_2_O_3_ series materials.

To investigate the generation of the B_2_O_3_, CN, and B_2_O_3_/CN series samples, thermo gravimetric analysis was performed from room temperature to 800 °C at a heating rate of 10 °C min^−1^ in N_2_ flow. As shown in Figure 8, B_2_O_3_ undergoes 6% weight loss from 68 °C to 335 °C. When the calcination temperature exceeded 335 °C, the thermogravimetric curve of B_2_O_3_ has a slight increase. The phenomenon may indicate that an exothermic reaction occurred that may have caused recrystallization. Youngjae et al. [33] reported that B_2_O_3_ forms a molten state after 325–450 °C, which increases the viscosity of the B_2_O_3_/CN system with increasing B_2_O_3_ content. Meanwhile, CN rapidly undergoes total weight loss from 580 °C to 725 °C, and is calcined to a volatile gas. In the composite materials, B1-CN and B3-CN undergo 6% weight loss from 50 °C; upon reaching the decomposition temperature of carbon nitride (580 °C), the curve shows a steep decline. On the other hand, B2-CN behaved uniquely among all the materials. It experienced a gradual weight loss of 15% from 50–360 °C, following which the weight loss rapidly increased at 578 °C, indicating that the structure of carbon nitride began to decompose. In the composite materials, the boron-oxygen band of boron oxide and the amino group (-NH_2_) of carbon nitride can interact in the molten state, which may weaken and break the chemical bonds between the melon unit and NH_2_ group. This was demonstrated by theoretical calculations, which indicated that the chemical bond between the melon unit and NH_2_ group was weakened after combination with the hydroxy group with an increase in temperature.

**Figure 9:**
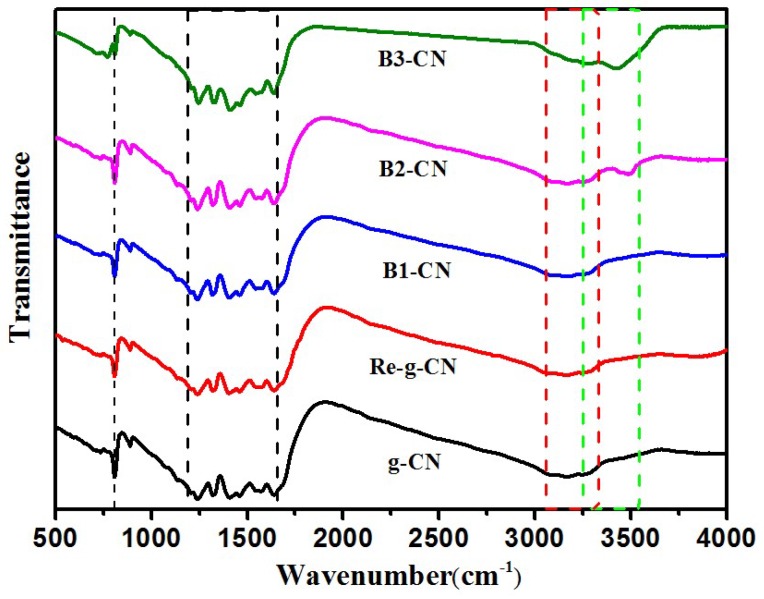
FT-IR spectra of CN, Re-CN, and CN/B_2_O_3_ series materials.

Figure 9 shows the Fourier-transform infrared (FT-IR) spectra of the CN and B_2_O_3_/CN series materials. In all samples, the multiple bands at 1200–1600 cm^−1^ are ascribed to the characteristic CN heterocycle stretching vibrations, and the sharp band at 810 cm^−1^ is assigned to the bending vibration of heptazine units [34]. This indicates that most of the structure of CN was retained after calcination and mixed with B_2_O_3_. The broad absorption band at around 3000–3300 cm^-1^ can be attributed to the stretching vibration of the NH bond, particularly the stretching vibrations of the primary and secondary amine groups [19,35]. The peak around 3200–3600 cm^−1^ in B2-CN and B3-CN can be assigned to OH [36].

### 3.2. Adsorption Property

**Figure 10:**
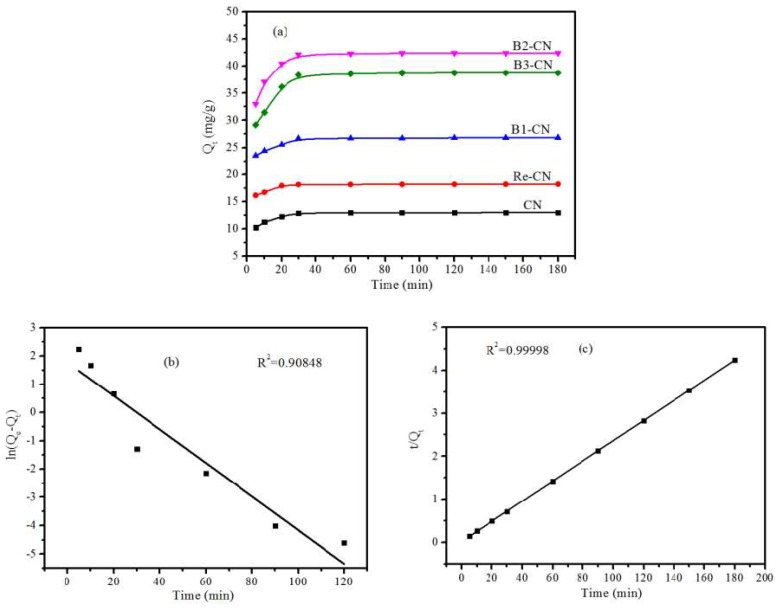
Adsorption kinetics curves for all samples (**a**); plots of the pseudo first order (**b**); plots of the pseudo second order (**c**).

To demonstrate the adsorption property of CN, Re-CN, and B_2_O_3_/CN series materials, the MB was chosen as the organic contaminant. Figure 10 is the MB adsorption kinetics curves for all samples. As shown in Figure 10a, CN has a low adsorption capacity, owing to the in-built NH, NH_2_ groups and π–π region, which provide basic sites on the surface that may be utilized for the removal of polluting molecules [37]. When the adsorbed get equilibrium, the adsorbed quantity of B2-CN has the highest adsorption efficiency which reached 43.11 mg/g, while that of CN was only 13.05 mg/g. This improved adsorption ability can be attributed to the calcination of CN in B_2_O_3_, yielding enhanced surface area and a large pore volume for pollution loading. In addition, boron doping in CN may extend the π-conjugation system, which is favorable for MB adsorption. Otherwise, to study the adsorption kinetics detail of B2-CN, the experimental data were fitted by pseudo first order kinetic model (Figure 10b) and pseudo second order kinetic model (Figure 10c). As shown in these figures, the correlation coefficients (R^2^) of the two pseudo order models were 0.90848 and 0.99998, respectively. Therefore, the adsorption of MB on B2-CN has a better correlation with the pseudo second order kinetic model than the pseudo first order kinetic model.

**Figure 11:**
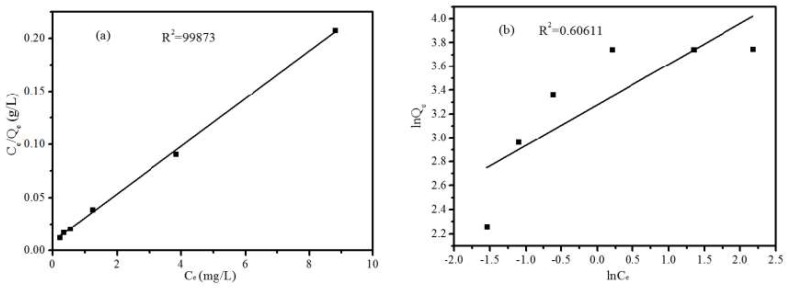
The linearized of Langmuir adsorption isotherm (**a**) and Freundlich adsorption isotherm (**b**) for MB adsorption by B2-CN.

Figure 11 shows the Langmuir adsorption isotherm and Freundlich adsorption isotherm for MB adsorption by B2-CN. The correlation efficient (R^2^) using the Langmuir model was 0.99873, which was much higher than the Freundlich model (R^2^ = 0.60611). This indicates that the adsorption of MB by B2-CN is attached to the monolayer adsorption, rather than multiple layers. With the increase of pollutant concentration, it is more conducive to the adsorption process. Therefore, it tends to approach the Langmuir adsorption isotherm model.

### 3.3. Photocatalytic and Recycling Efficiencies

**Figure 12:**
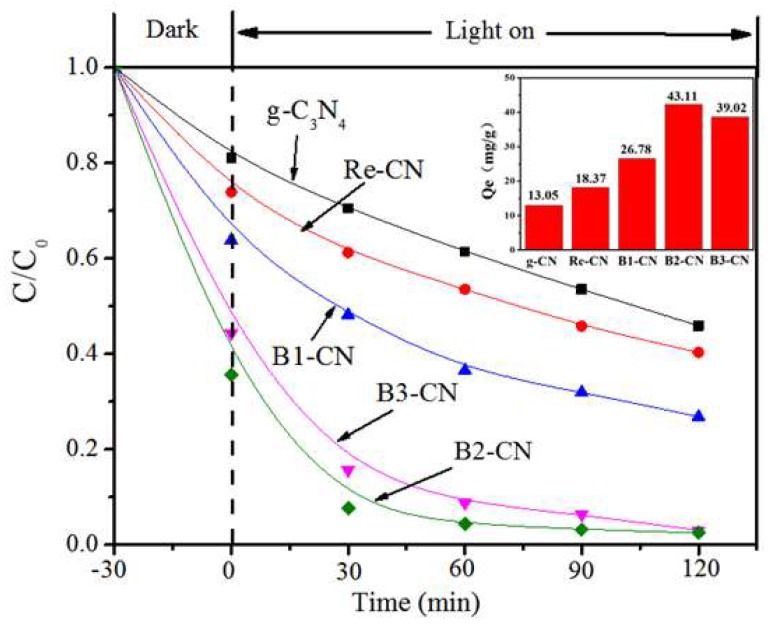
Removal efficiency and adsorption capacity (insert) of MB for all samples.

Figure 12 shows the dark adsorption and light-driven photocatalytic reduction of MB over the CN, Re-CN, and B_2_O_3_/CN series materials in an aqueous suspension. In the dark adsorption experiment, adsorption equilibrium was achieved after 30 min of magnetically stirring the mixture of the photocatalytic material and MB solution. After combining the two materials, the graph (Figure 12, inset) clearly shows that the adsorption ability of all B_2_O_3_/CN materials is much higher than those of CN and Re-CN. This adsorption ability of B_2_O_3_/CN toward MB may be closely related to B_2_O_3_. In 100 mL of the 20 mg/L MB solution, the adsorbed quantity of B2-CN was 43.11 mg/g, while that of CN was only 13.05 mg/g. After the dark experiment, the mixed MB solution was irradiated using a xenon lamp (λ > 400 nm) up to 2 h for the photocatalytic studies. After dark reaction and visible light irradiation, the highest removal efficiency obtained with B2-CN was in excess of 95% in 1 h. The degradation mechanism of MB was the same as that of general organic pollutants by photocatalysis. Degradation involves the generation of e^-^ and h^+^, which again generate O^2^ and OH radicals, respectively. These radicals are highly reactive and act as degrading agents.

**Figure 13:**
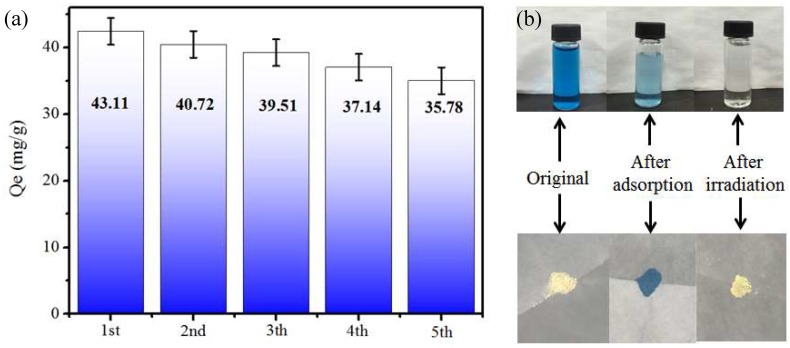
(**a**) cycling runs for the adsorption capacity of MB over B2-CN; (**b**) the MB solution and sample color in the original, after adsorption, and after irradiation stages.

**Figure 14:**
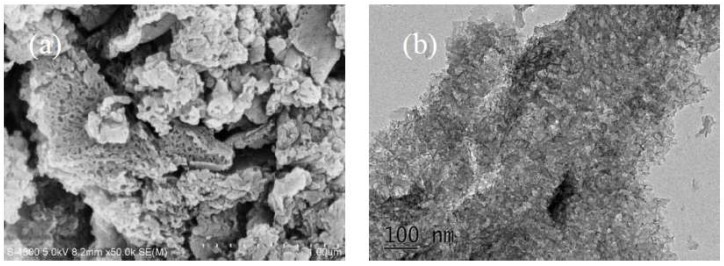
The SEM (**a**) and TEM (**b**) micrographs of B2-CN after the adsorption-photocatalytic process.

Lastly, to evaluate the stability of CN/B_2_O_3_ for reutilization in practical applications, B2-CN was subjected to five successive adsorption and degradation cycles, following which the photocatalytic materials were collected from the MB solutions. The adsorbed saturated “blue” powders were then dissolved in water for xenon light irradiation. After 1 h of irradiation, the adsorbed saturated “blue” materials regained their original light yellow color (Figure 13b). After the adsorption–degradation experiment, the morphology of the material was observed by SEM and TEM (Figure 14). We found that the surface and pores of the material have some dilapidation, but the original morphology was basically retained. In the fifth run, the adsorption capacity of MB decreased from 43.11 mg/g to 35.78 mg/g, which could be due to the loss of the catalyst or holes blockage. However, the efficiency decreased only 17.01% after five cycles, demonstrating that B2-CN has excellent stability and potential for practical applications.

**Figure 15:**
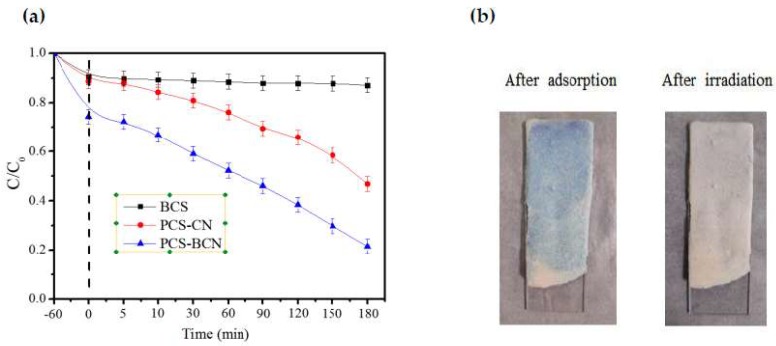
Photocatalytic of MB removal efficiency of BCS, PCS-CN and PCS-BCN under dark adsorption and light-driven photocatalytic (**a**) and color changes following irradiation of MB on the PCS-BCN surface (**b**).

Figure 15a describes the MB removal efficiency of BCS, PCS-CN and PCS-BCN under dark adsorption and light-driven photocatalytics. The result of experiments showed that the photocatalytic materials added in the concrete could improve the ability of photocatalytic substrate effectively. Therefore, PCS-BCN obtained better adsorption and photocatalytic results in the experiment, which was consistent with powder material experiment. Moreover, in order to explore the regeneration of PCS-BCN, we placed the substrates under xenon lamp for irradiation. After 1 h of irradiation, the “blue” concrete substrate surface regained the original state (as shown in Figure 15b). Therefore, the experiment result proves that the photocatalytic concrete has a good effect on adsorption-catalysis and regeneration, which has a certain applied research value in engineering applications for surface coating.

## 4. Conclusions

In summary, adsorption–degradation-functionalized CN was successfully prepared using B_2_O_3_ and CN by calcination. Compared with pristine CN, B-CN exhibited higher specific surface area and particle size. Results of the adsorption–degradation experiments indicated that the removal efficiency of B-CN for MB was higher than that of pristine CN, and its adsorption capacity was 43.11 mg/g, nearly 3.3 times greater that of pristine CN. The MB adsorption process of B2-CN was described using the Langmuir model and pseudo second order kinetic model. Otherwise, B-CN added into concrete also exhibits good performance and regeneration ability, which are very important characteristics for practical applications. Thus, this study provides a new strategy to develop adsorption–degradation-functionalized CN.

## Figures and Tables

**Table 1:** BET surface area, average pore size, and pore volume of CN, Re-C_3_N_4_, and CN/B_2_O_3_ series composite materials.

Sample	CN	Re-CN	B1-CN	B2-CN	B3-CN
BET surface area (m^2^∙g^−1^)	10.358	14.311	15.499	37.049	23.859
average pore size (nm)	8.979	9.467	9.049	17.682	13.162
average pore volume (cm^3^∙g^−1^)	0.023	0.034	0.035	0.105	0.094

## Abbreviations

Carbon nitride (CN), methylene blue (MB), B_2_O_3_/CN (B-CN), B1-CN (CN/B_2_O_3_ = 3:1), B2-CN (CN/B_2_O_3_ = 1:1), B3-CN (CN/B_2_O_3_ = 1:3), X-Ray diffraction (XRD, diffuse reflectance spectra (DRS), X-ray photoelectron spectroscopy (XPS), scanning electron microscope (SEM), transmission electron microscope (TEM).
